# The Application of Mechanical Stimulations in Tendon Tissue Engineering

**DOI:** 10.1155/2020/8824783

**Published:** 2020-09-24

**Authors:** Renwang Sheng, Yujie Jiang, Ludvig J. Backman, Wei Zhang, Jialin Chen

**Affiliations:** ^1^School of Medicine, Southeast University, 210009 Nanjing, China; ^2^Department of Integrative Medical Biology, Anatomy, Umeå University, SE-901 87 Umeå, Sweden; ^3^Jiangsu Key Laboratory for Biomaterials and Devices, Southeast University, 210096 Nanjing, China; ^4^China Orthopedic Regenerative Medicine Group (CORMed), China

## Abstract

Tendon injury is the most common disease in the musculoskeletal system. The current treatment methods have many limitations, such as poor therapeutic effects, functional loss of donor site, and immune rejection. Tendon tissue engineering provides a new treatment strategy for tendon repair and regeneration. In this review, we made a retrospective analysis of applying mechanical stimulation in tendon tissue engineering, and its potential as a direction of development for future clinical treatment strategies. For this purpose, the following topics are discussed; (1) the context of tendon tissue engineering and mechanical stimulation; (2) the applications of various mechanical stimulations in tendon tissue engineering, as well as their inherent mechanisms; (3) the application of magnetic force and the synergy of mechanical and biochemical stimulation. With this, we aim at clarifying some of the main questions that currently exist in the field of tendon tissue engineering and consequently gain new knowledge that may help in the development of future clinical application of tissue engineering in tendon injury.

## 1. Introduction

Mechanical stimulus has a huge impact on life activities, which is evident in gene expression, cell life activities, functions of living systems, and individual growth and development. With the redistribution of body fluids and the reduction of skeletal load under weightless conditions, bone loss and increased calcium secretion occur to the bones, which seriously affect the function of the musculoskeletal system [1, 2]. In the context of induced differentiation of stem cells, different types of mechanical stimulations may play different roles. For instance, mechanical stretching has been widely used in tendon tissue engineering to induce tenogenic differentiation, while mechanical compression is beneficial for osteogenic differentiation as well as for chondrogenic differentiation [3–5].

Mechanical stimulation plays a significant role in many aspects of tendon tissue engineering. Applying mechanical stretching to engineered tendons could promote cell infiltration and proliferation [6, 7], induce the extracellular matrix (ECM) deposition and the collagen fiber alignment [6, 8, 9], and also activate mechanically sensitive receptors which subsequently promote tenogenic differentiation [10–12]. In addition, magnetic force could be used as a mechanical stimulus to reduce the formation of fibrous scar tissue and regulate inflammatory responses [13]. Nowadays, mechanical stimulations have been widely used in tendon tissue engineering. However, the optimal regimes of mechanical stimulation for different stem cells for tendon tissue engineering are not yet clarified, nor their inherent mechanisms of mechanical transduction.

In this review, we will make a retrospective analysis of the past decades in the field of applying mechanical stimulation in tendon tissue engineering, as well as the inherent mechanisms. We will also propose some of the most promising directions of mechanical stimulation in tendon tissue engineering. Due to the high similarity in structure and function, tendon and ligament are often discussed undividedly. Therefore, the term tendon is related to both tendon and ligament in this review.

## 2. Tendon and Tendon Repair

### 2.1. Tendon Structure

As a highly specialized load-bearing structure, the tendon has an indispensable role in the force transmission between the muscle and bone, thus the tendon is vital for the muscle function and tolerates much higher strain as compared to the muscle belly [14, 15]. Tendons consist of dense regular connective tissues made up of multiple collagen fibrils forming collagen fibers with the paralleled arrangement along the direction of the strain [13]. The tendon tissue ECM is mainly composed of collagen (60%-85% of dry weight) of which approximately 90% is collagen I (COL I) and 10% is collagen III (COL III) and the remaining ECM consist of proteoglycans (1-5% of dry weight). Only few tenocytes, progenitor cells, are located between the collagen fibrils. The sparse vascular supply is either from the related muscle or bone or from the tissue surrounding the tendon, and the nerve innervation is mainly found in the surrounding tissue [15–17]. The hypocellular and the hypovascular natures of tendons determine their poor self-healing capacity after injury [18–20].

### 2.2. Tendon Repair

Tendon injury is the most widespread musculoskeletal disease, especially the Achilles, patellar, and rotator cuff tendons [20, 21]. Injury to these tendons accounts for more than 30% of all musculoskeletal conditions for which people seek help within the primary healthcare system, and 30 million surgical procedures are performed annually worldwide [22, 23]. Unfortunately, it is almost impossible for tendons to be repaired perfectly due to the poor autonomous healing capability, which frequently results in persistent symptoms and reinjury. At present, tendon injuries are usually treated by conservative or surgical approaches. Conservative treatments include drug injection such as corticosteroids and nonsteroidal anti-inflammatory drugs (NSAIDs), low-intensity shock wave, ultrasound, and mechanical loading including eccentric training. All these treatment strategies require a long time for the tendon to recover, and the results are often not satisfactory with symptoms that often recur [18, 22, 23]. Surgical treatments such as suture, autograft, allograft, and xenograft also have some disadvantages, including the low availability of grafts, donor site morbidity, infection risk, and inflammatory response [18, 23, 24]. Some biological therapies, such as gene therapy, growth factor therapy, and stem cell therapy, have made great progress over the years by scientists, but most of them are still in the stage of *in vitro* or animal testing; thus, further research is required for subsequent clinical trials [19, 25]. In summary, at present, there is no optimal treatment strategy for tendon injury. New and effective treatments are in urgent need to be developed.

### 2.3. Tendon Tissue Engineering

Tendon tissue engineering aims at constructing engineered tendons with similar properties to natural tendons and finally to replace damaged tendons by surgery. Commonly, stem cells are seeded in scaffolds and then cultivated in an environment with appropriate growth factors and/or biomechanical stimulation, aiming at constructing an ideal tissue-engineered tendon. Based on the principles of tissue engineering, there are three strategies to optimize the engineered tendon: selecting the ideal cells, improving the properties of the scaffold, and providing appropriate growth factors and/or biomechanical stimulation [26]. Various stem cells have been applied in tendon tissue engineering. For instance, bone marrow mesenchymal stem cells (BMSCs) [27, 28], adipose stem cells (ASCs) [29], and tendon-derived stem cells (TDSCs) [6, 10, 15]. The cell type most commonly applied for tissue engineering is BMSCs, which are multipotent stem cells isolated from bone marrow with self-renewal capability, multilineage differentiation potential, and immune system tolerability [30, 31]. ASCs are stem cells derived from adipose tissue with fantastic differentiation and migration capacities, and they seem to be quite appropriate for tendon therapy [32]. TDSCs have been isolated from tendons of different organisms, and they have similar self-renewal and multilineage differentiation capacities as the BMSCs but have a higher expression level of tendon-related genes [6]. Therefore, TDSCs are potentially the ideal cells to use for tendon tissue engineering. Nowadays, synthetic materials like polylactic acid and polyglycolic acid have been applied to create scaffolds for tendon tissue engineering [6, 33, 34]. However, researchers are also keen to find out the excellent biomaterials, such as collagen [35, 36], silk fibroin [36], alginate, and gelatin [23]. In addition, the structure of the scaffolds is also important to optimize. It is known that a few hundred-micron pore sizes and a porosity of over 90% in the scaffold material facilitate cell infiltration [37]. Scaffolds made of aligned fibers with wavy morphology exhibit excellent mechanical properties and the effects in promoting cell proliferation, infiltration of cells in-between the fibers, and stimulating tenogenic differentiation [38–41]. In addition, various growth factors have also been shown to stimulate tenogenic differentiation and tendon regeneration such as connective tissue growth factors (CTGF), transforming growth factors *β* (TGF-*β*), and growth differentiation factors (GDF) [42]. Although many studies on tendon tissue engineering have been conducted in the past decades, there still exist some major challenges, such as the comprehensive consideration of the mechanical properties of the scaffold and its integration with cells. Optimal biomechanical stimulation during tendon tissue engineering may improve the construct by regulating the remodeling of ECM and promotion of cell infiltration [7], alignment [6], proliferation [7], and differentiation [35].

## 3. Main Strategy of Mechanical Loading in Tendon Tissue Engineering

**Figure 1:**
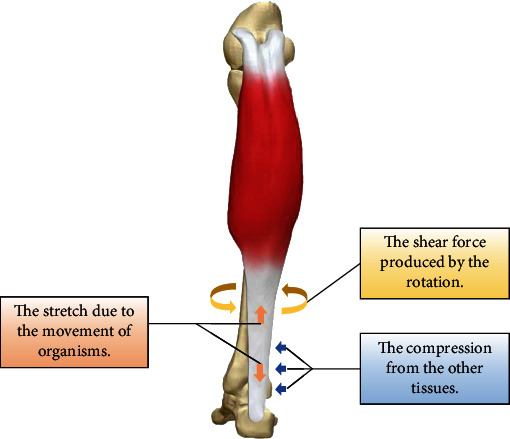
Biomechanical stimulations in natural tendon. Three arrows with different colors indicate three types of biomechanical stimulations including stretch, shear force, and compression.

Biomechanical signals are involved in the growth and development of organisms, which can stimulate and induce tissue formation. Biomechanics is a branch of biophysics, which applies the principles and methods of mechanics to the quantitative research of biomechanical problems in for example blood, body fluids, organs, and bones. The physiological mechanical stimulus on the tendon is comprised of tensile strain, shear force, and compression (Figure 1) [10]. Due to the main function of tendon, the mechanical stretching caused by tension is the main mechanical stimulus throughout the growth and development of tendons. Therefore, it is reasonable to provide mechanical stretching for the construction of engineered tendons to mimic the natural microenvironment of tendons. In tendon tissue engineering, dynamic and static stretching is currently the most widely used mechanical stimulation *in vitro*[11, 20, 43]. Natural mechanical stimulation *in vivo* has also been applied to tendon tissue engineering with great significance.

### 3.1. Dynamic and Static Stimulation *In Vitro*

Mechanical stretching is the main strategy to achieve mechanical loading in tendon tissue engineering, which could be divided into dynamic and static mechanical stretching [11, 20, 43]. Appropriate mechanical stretching is beneficial to the formation of engineered tendons as it regulates cell behaviors and tissue remodeling [6, 7, 24, 35]. However, mechanical stretching can also have negative effects. For instance, mechanical stretching may increase the diameter of the scaffold, make it elongated, and decrease its Young's moduli [23, 35], i.e., reducing the mechanical properties of the scaffold. Also, excessive mechanical stretching will result in early differentiation and apoptosis of stem cells [23, 43]. The applications of mechanical stretching during the past decades are summarized in Table 1.

Dynamic stretching is the most commonly used type of mechanical stretching stimulus and can be regulated by three main parameters, which are (1) strain, (2) frequency, and (3) rest interval [11]. Different protocols usually bring different and even opposite effects to tenogenic differentiation and tendon tissue engineering.

1. Strain. It has been reported that mechanical stretching can not only induce tenogenic differentiation but also promote osteogenesis, adipogenesis, and chondrogenesis, which is closely related to the percentage of strain [10, 18, 59]. Thus, it could be speculated that only mechanical stretching of a certain range of strain can induce tenogenic differentiation. However, researchers have reached different, or even opposite conclusions. For example, Chen et al. found that the lower strain (3%) promoted osteogenic differentiation, while the higher strain (10%) upregulated the expression of tendon and ligament-related genes [18]. However, Patel et al. found that a 4% strain promoted the tendon differentiation of TDSC, while an 8% strain might induce osteogenesis, adipogenesis, and chondrogenesis [59]. Actually, the reported strains that could promote tendon differentiation range from 1% to 15% (Table 1). According to the physiological strain of the tendon *in vivo*, the strain of dynamic stretching should be 4%-8% (at most 10%) [60]. Zhang et al. and Rinoldi et al. both applied dynamic stretching with a 15% strain in their studies. Although it promoted expression of tendon-related genes such as *Scleraxis* (*SCX*) and *Tenascin-C* (*TNC*), it resulted in lower protein expression of COL I and TNC [23, 35]. In addition, Nam et al. found that human BMSCs had the highest expression of tendon-related genes and proteins at strain conditions of 8% and 12%, and the latter reached its peak faster [27, 28]. Therefore, a strain of 1%–12% seems to be a broad range that could be applied in tendon tissue engineering. Generally, a too high strain may cause early cell differentiation and apoptosis and may also reduce the mechanical properties of the scaffold, such as excessive elongation or increased pore size [23, 35]. On the other hand, a too low strain may not have the expected stimulatory effects. Due to different loading methods and loading systems (such as duration, tissue fixation methods, and stem cell types), the optimal strain varies and should thus be optimized for each specific condition [18]
2. Frequency. Most of the stretching frequency applied in tendon tissue engineering is not higher than 1 Hz (Table 1). Some studies found that 1 Hz of mechanical stretching may be the best condition to induce various cellular responses including a high level of cell proliferation and tenogenic differentiation [11, 27]. However, Engebretson et al. demonstrated that the lower frequencies are better for improving the quality of engineered tendons, while the positive effect of stimulus would decrease when it is over 1 cycle/min (0.017 Hz). They found that the lower frequency and shorter duration (1 cycle/min and 0.5 or 1 h/day) were more likely to promote the production and alignment of COL I fibers and cell proliferation as compared to higher frequency and longer duration [43]. Generally, lower frequencies (below 1 Hz) are beneficial for cell proliferation and tenogenic differentiation. The optimal stretching frequency from each study is different, probably due to the differences in bioreactors, cell types, and other stretching parameters. Mechanical stretching with higher frequency (higher than 1 Hz) influences cell proliferation and reduces the expression of ECM proteins in tendons [23, 43], and it may also induce apoptosis, which can explain why cell proliferation decreases [43]
3. Rest Interval. Cells gradually adapt to the stimulus, thereby a reduction in the effect of applied mechanical stimulus [11]. By adding rest interval, the mechanical sensitivity of the cells can be restored, and ultimately more positive effect can be achieved [61]. As mentioned earlier, Engebretson et al. found that groups with shorter duration and lower frequency had higher levels of cell proliferation. The highest proliferation was found in the group with mechanical stretching 0.5 hour/day and with 1 cycle/minute, which resulted in an increase of 203% as compared to the static control. Mechanical stretching lasting longer than 1 hour/day would limit its beneficial effects due to adaptation to the stimulus [43]

**Figure 2:**
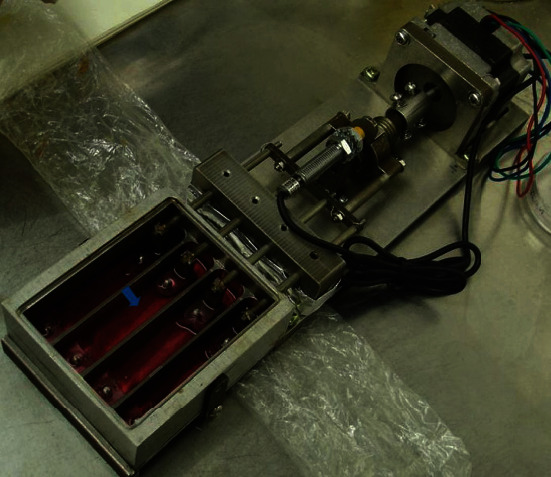
A custom-made bioreactor. Engineered tendon (cell-seeded scaffolds) is placed within each chamber (blue arrow), with two ends anchored. Amplitude and frequency of the bioreactor could be set.

In general, the effects of various parameters (range of strain, frequency, rest, and duration) of dynamic stretching on tenogenic differentiation are significant but it is difficult to distinguish whose impact is most efficient. To evaluate the role of each parameter of dynamic stretching in tendon tissue engineering, a bioreactor capable of regulating different parameters at the same time is essential. A bioreactor can provide suitable biomechanical and biochemical stimulus to the engineered tendon constructs, mimicking the microenvironment of natural tendons. The activating system and the culture chamber are the main components of bioreactors. In addition, other systems can be added to achieve circulation and in-depth analysis of the medium. Presently, the LigaGen system (http://www.tissuegrowth.com) and the Bose® ElectroForce® BioDynamic® system (http://www.bose-electroforce.com) are the well-developed commercially available bioreactor systems. Both systems can provide precise and programmed mechanical stretching. The LigaGen system can detect the stiffness of the sample in real-time and adjust the instrument itself according to different requirements. The Bose® ElectroForce® BioDynamic® system can monitor sample strain and perform biomechanical tests in real-time [62]. In addition, various bioreactors are also developed by different research groups to meet their own individual specific requirements, and some of them showed a good performance in constructing engineered tendons (Figure 2) [58, 60, 63].

Some earlier studies also reported the positive effect of static mechanical stimulation on tenogenic differentiation (Table 2). Dynamic stretching with higher frequency and longer duration decreases the level of cell proliferation [43]; comparably, it was found that continuous application of static stretching will reduce the mechanical sensitivity and thus the proliferation of cells. In addition, long duration of static stretching will reduce the total tension to the cells for two reasons; (1) due to adaptation to the stimulus and (2) all newborn cells will not sense the stretching [23]. Therefore, currently, more and more studies in this field have focused on the optimization of protocols for dynamic stretching, instead of static mechanical stimulation.

### 3.2. 2D and 3D Loading Models *In Vitro*

At present, two-dimensional (2D) loading models and three-dimensional (3D) loading models *in vitro* have been applied to the research of tendon mechanobiology. In 2D loading models, cells are usually seeded on a sheet and receive mechanical stretching indirectly by stretching the sheet [10]. In this model, mechanical stretching can be accurately transmitted to the cytoskeleton, and the relationship of biological response and mechanical stimulation can be studied [68]. Both uniaxial and biaxial stretching have been applied to 2D loading models and have been shown to influence the promotion of tenogenic differentiation [68]. However, there are some disagreements regarding stem cell differentiation with the application of biaxial stretching. Wang et al. found that uniaxial loading promoted tenogenic differentiation but biaxial loading induced chondrogenic, adipogenic, and osteogenic differentiation of TDSCs [10]. However, some other researchers have shown that biaxial loading could also promote tenogenic differentiation [8, 69–71]. Biaxial loading provides multidirectional stretching, including longitudinal and transverse or circumferential directions, which is different from the physiological mechanical environment of tendon cells [68]. Therefore, stem cells may simultaneously show the higher expression of multiple tissue genes under biaxial stimulation, and the differences in gene expression levels may be caused by different loading conditions and cell types.

Even though the 2D loading model can be used to investigate the effects of mechanical stimulus on cells, it cannot replace the significance of 3D loading models since the effect of mechanical stretching on 3D engineered tendon constructs are influenced by many factors such as pore size, topography, and the material of the scaffolds [68]. The 3D loading models are constructed by seeding cells in a 3D material, which transfers stretching to the embedded cells. The effect and involved signaling pathways of the same stretching protocol could differ dramatically between a 2D loading system and a 3D loading system. For instance, Wang et al. found that osteogenesis and adipogenesis differentiation was promoted by 2D uniaxial loading, but inhibited by 3D uniaxial loading using the same mechanical stretching protocol [10]. Connexin 43 is a gap junction protein that mediates intercellular communication. Wang et al. discovered that 2D-loaded cells expressed more connexin 43 when uniaxially loaded; however, the opposite results were obtained in 3D loaded tendons. This indicates that under 2D conditions, cells perceive mechanical stimulation through cell body junctions, while under 3D conditions, cells perceive mechanical stimuli through cell-ECM interactions. It has been found that mechanical loading under 3D conditions can promote tenogenic differentiation and tendon ECM remodeling which facilitate the construction of engineered tendon[6, 11, 23, 24]. Compared with the 2D loading system, the 3D system more closely simulates the physiological mechanical loading of natural tendons. Therefore, the 3D loading model is more relevant for tendon tissue engineering.

### 3.3. Natural Mechanical Stimulation *In Vivo*

**Figure 3:**
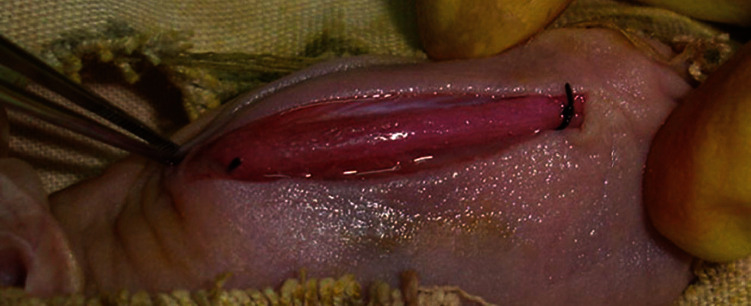
Engineered tendon transplanted into the back of a nude mouse to exert physiological mechanical stimulation. The construct is sutured to the fascia, which receives mechanical stretching caused by the natural movement of the mouse back. Reprinted from Biomaterials (2010), Vol. 31, Chen JL, et al., Efficacy of hESC-MSCs in knitted silk-collagen scaffold for tendon tissue engineering and their roles, Pages 9438-9451, Elsevier (2010), with permission from Elsevier.

The long-term goal is that tissue-engineered tendons eventually will be used clinically; therefore, its construction and functionality *in vivo* are of great significance. Some groups transplanted engineered tendons into the knee joints [8, 57] and backs [58] of miniature pigs or mice to give them physiological mechanical stimulation (Figure 3), which is caused by the movement of the recipient animals. These natural mechanical stimulations induced the formation of a more mature tendon-like tissue by promoting the tenogenic differentiation of stem cells, inducing a physiological cell shape and arrangement, and promoting the deposition and arrangement of tendon ECM [6, 57, 58]. Juncosa-Melvin et al. showed that the maximum stress of engineered tendons cultured *in vivo* increased by 3000 times after 2 weeks, which could not be accomplished in any current bioreactors *in vitro* [62, 72]. Therefore, the natural mechanical stimulation has great potential if applied to tendon tissue engineering. Interestingly, Zhang et al. and Xu et al. prestretched engineered tendon *in vitro* before applying natural mechanical stimulation *in vivo*. The prestretched group was found to have more deposition of aligned tendon ECM, as compared to the unstretched [6, 35]. The reason for the positive effect might be because the prestretching *in vitro* promotes the integration of seeded cells and the scaffold, thus show better stimulatory effects after transplantation. In conclusion, natural mechanical stimulation and the combination of mechanical stretching *in vitro* and *vivo* help the formation of matured engineered tendons, which are of great significance for tendon tissue engineering.

## 4. Effect of Mechanical Loading on Tendon Tissue Engineering

Mechanical stimulation has been shown to promote the tenogenic differentiation of stem cells and the deposition of tendon ECM, thus improving the properties of engineered tendon constructs. Until now, different mechanical stimulation protocols have been reported in tendon tissue engineering to mimic the mechanical environment of tendons under natural conditions. Some previous studies have demonstrated that mechanically stimulated tissue-engineered tendon shows more promising results in tendon repair and regeneration *in vivo.* For instance, Xu et al. evaluated the repair effect of mechanical stimulated engineered tendons in a rabbit patellar tendon defect model. They found that the repaired tendons in the experimental group exhibited more and aligned collagen fibers, aligned spindle-shaped healing tenocytes, and significantly increased ultimate stress and Young's modulus, as compared to those in the control group [6]. Lee et al. applied the mechanically stimulated engineered tendon to porcine anterior cruciate ligament (ACL) reconstruction *in vivo* and found that the ultimate tensile load of the repaired tendons improved significantly (within 80% of the native porcine ACL) after three months postsurgery, with higher matrix synthesis and increased stiffness, as compared to the tendons repaired by the nonmechanical-stimulated engineered tendon [8]. Furthermore, some studies discovered that engineering tendon with mechanical loading *in vivo* exhibited more mature collagen fibrils, better-aligned collagen fibers, and bigger tissue volume with improved mechanical properties, as compared to the loading *in vitro* and the nonloading *in vivo*[6, 73, 74]. In general, there are two explanations of how mechanical stimulation promotes tendon repair and regeneration.

1. Mechanical stimulation promotes the tenogenic differentiation of stem cells. The gene expression of tendon markers and the synthesis of tendon ECM are two important outcomes to measure tenogenic differentiation. As tendons have no specific markers, expressions of several important tendon-related markers are usually detected, such as Scleraxis (SCX), Mohawk (MKX), and Tenomodulin (TNMD) [9, 23]. SCX is a known early transcription factor expressed in tendon progenitor cells and tenocytes [75, 76]. MKX is recognized as a transcription factor expressed in developing tendons [76]. And TNMD is a tension-regulating protein, which is related to the phenotype of tenocytes and is considered as a late marker of tendon formation [7]. As shown in Tables 1 and 2, mechanical stimulation can promote the expression of these important tendon-related markers. COL I is the main component of tendon ECM, therefore promoting the expression of COL I can be considered, to some extent, as a marker of tenogenic differentiation of stem cells. In addition, other ECM molecules such as COL III, decorin (DCN), Tenascin-C (TNC), N-cadherin, elastin (ELN), and fibronectin (FN) are other components of the tendon, and therefore their expressions were evaluated as well in many reports [7, 28, 51]. However, all those ECM molecules could also be found in many other tissues. For example, the tissues expressing COL I include tendons, basement membranes, and skin and blood vessels [77]. Based on the fact that there is not any unique ECM molecule only found in tendons, it is not enough to evaluate tenogenic differentiation by only using the expression of certain ECM molecule
2. Mechanical stimulation regulates cell behaviors and improves the mechanical properties of engineered tendons by remodeling of the ECM. It has been found that mechanical stimulation promotes cell proliferation [6, 24], migration [23], infiltration [6, 8], and alignment [6], as well as ECM deposition [7, 24], which are all of great significance for successful construction of engineered tendons. For instance, Xu et al. cultured the TDSC-seeded poly(L-lactide-co-*ε*-caprolactone)/collagen construct under dynamic stretching for tendon tissue engineering. They found that mechanical stimulation induced an increased proliferation and a similar morphology with tenocytes, and finally it increased the expression of tendon-related ECM genes and proteins, which resulted in significantly improved mechanical properties of the engineered tendon (about 52% of Young's modulus and 60% of ultimate tensile stress of the nature tendon) [6]

## 5. Signal Transduction of Mechanical Loading in Tendon Tissue Engineering

Substantial progress has been made in the study of signal transduction following mechanical stimulation. Cells sense and deliver mechanical stimulation through cell adhesion molecules (CAMs) [78]. CAMs are dynamically connected to the cytoskeleton, responding to mechanical tension and transmitting stimulus to the nuclear membrane, which in turn triggers changes in cellular gene expression [78]. Since there are only a few cells in both natural and engineered tendons, the transmission of mechanical stimulus in tendon tissue engineering is more likely to depend on the interaction between cells and ECM by CAMs [10], which is a mechanism that should be further explored in order to construct engineered tendons successfully.

**Figure 4:**
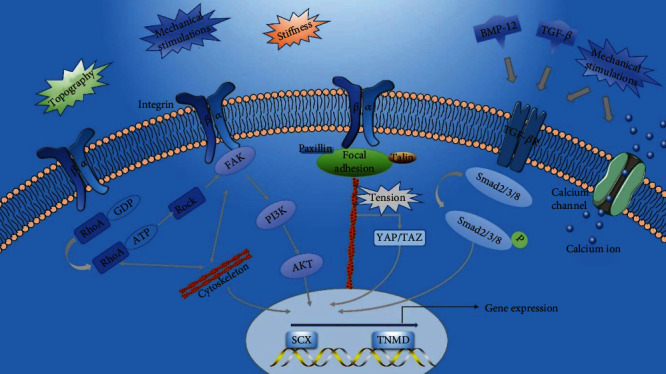
Signal transduction of mechanical loading in tenogenic differentiation. The mechanical stimulations and the mechanical properties of substrate including topography and stiffness can activate the integrins, which causes the changes of downstream signaling molecules and results in tenogenic differentiation. The mechanical stimulations and growth factors both can activate the TGF-*β* receptors, inducing tenogenic differentiation. The mechanical stimulations can open the calcium channel and bring about the inflow of Ca^2+^, which regulates tenogenic differentiation.

It has been reported that the signal transduction network consists of focal adhesion kinase (FAK) [11, 12], phosphatidylinositol 3-kinase/protein kinase B (PI3K/AKT) [10, 79], Rho proteins/Rho-associated protein kinase (RhoA/ROCK) [12], yes-associated protein/transcriptional coactivator with PDZ binding (YAP/TAZ) [13], and Smad [10, 80]. All of these signal transducers are involved in tenogenic differentiation induced by mechanical stimulation (Figure 4). There are also some mechanically sensitive receptors on the cell membrane, such as integrins, growth factor receptors, and stretch-activated ion channels [78]. Transmembrane integrins connect ECM proteins and focal adhesion proteins, and the latter are connected to the nuclear membrane through the cytoskeleton (actin fibers). Thus, integrins can transmit forces across the nuclear membrane and mediate the response of mechanically loaded cells [13]. Besides, integrins can also detect the stiffness (elasticity), topography, and surface chemistry of the matrix [81]. These detections by the integrins can activate FAK and RhoA and thereby induce changes in downstream signal molecules [81]. PI3K/AKT pathway has been demonstrated to participate in the regulation of tenogenic differentiation as the downstream pathway of FAK [10, 79, 81]. For example, Wang et al. and Cong et al. found that when the PI3K/AKT pathway was inhibited, tenogenic differentiation and the formation of engineered tendons are weakened [10, 79]. Therefore, although the PI3K/AKT pathway has also been reported to be associated with osteogenic differentiation of stem cells, it does play an important role in the tenogenic differentiation induced by mechanical stimulation. RhoA/ROCK pathway is another downstream signaling pathway of mechanical loading. RhoA is a member of the small G protein superfamily and can activate downstream ROCK. RhoA/ROCK is a downstream molecule of integrins, which together with FAK regulates mechanical stretch-induced cytoskeletal reorganization [81]. Xu et al. discovered that RhoA/ROCK affected FAK activation and coregulated the formation and rearrangement of actin fibers, thereby inducing tenogenic differentiation. At the same time, the cytoskeleton appeared to regulate its changes through feedback [12]. Besides, Tomás et al. demonstrated that changes in cytoskeleton tension following mechanical stimulus can activate YAP/TAZ in the cytoplasm to be transferred into the nucleus and promote the expression of tendon markers *SCX* and *TNMD* [13]. The TGF-*β*/Smad pathway is recognized as the most relevant pathway to tendon differentiation [82, 83]. It has been reported that mechanical stimulation and growth factors like TGF-*β* and BMP-12 (GDF-7) can activate growth factor receptors such as the TGF-*β* type I/II receptor. These receptors can contribute to the activation of the downstream Smad 2/3/8 pathway which promotes tendon formation [10, 80]. In addition, mechanical stimulation can activate mechanically sensitive ion channels, leading to the influx of cations (such as Ca^2+^), thereby inducing some cellular responses including the transmission of intracellular signal, actin polymerization, and cytoskeletal remodeling [11, 78, 84].

## 6. Promising Directions of Mechanical Stimulation in Tendon Tissue Engineering

### 6.1. Magnetic Force Stimulation

A previous study has demonstrated that magnetic stimulation has improved the biological performance as compared to the equivalent nonmagnetic mechanical stimulation in tendon tissue engineering [13]. Magnetic stimulation usually includes two aspects: the action of the magnetic field and the indirect mechanical force produced by a magnetic field upon magnetic particles (MNPs).

A low-frequency magnetic field has been applied to regulate the inflammatory response in tendon treatment [84]. Furthermore, Pesqueira et al. demonstrated that low-frequency static magnetic field promoted the expression of tendon-related genes (*SCX*, *COL1A1*, *COLA3*, *TNC*, and *DCN*) by regulating the intracellular calcium ion concentration and activation of oxygen release, and that the effect was related to the duration of exposure [84]. Hence, applying magnetic field in tendon tissue engineering has great potential.

Interestingly, the studies of magnetic force upon MNPs also have shown promising results in tendon tissue engineering [13, 80, 85, 86]. In biomedicine, MNPs have been used to label, track, and promote the life activities of stem cells such as proliferation, migration, and differentiation [86]. MNPs have two applications in tendon tissue engineering. (1) The direct application of MNPs alone. MNPs can be cocultured with stem cells. The mechanical force generated by the magnetic field will be transmitted to the stem cells, thus promoting their tenogenic differentiation. Gonçalves et al. labeled the human ASCs with chitosan-encapsulated MNPs to construct magnetically functionalized cells, which can be subjected to indirect and adjustable mechanical stimulation by applying a magnetic field [85]. In another study, they attached MNPs to antibodies (activin), which made the MNPs specifically bind to the RctRIIA (mechanically sensitive receptor). When supplied with a suitable magnetic field, the RctRIIA was remotely activated, which resulted in the activation of the Smad 2/3 pathway and triggered a tendon-related transcription response [80]. Both of these attempts can effectively deliver mechanical stimulation to cells and induce tenogenic differentiation without relying on scaffolds, which therefore avoids the possible negative effects of mechanical stimulation on scaffold properties such as increased pore size, elongation, and decreased elasticity. In addition, it can provide regular mechanical stimulation for the engineered tendon after transplantation, which may play a positive role in promoting tendon repair and regeneration. (2) The application of MNPs incorporated scaffolds. MNPs can be used to fabricate magnetically responsive scaffolds. MNPs in the scaffold can vibrate in response to an external magnetic field, which deflects the material to produce a transient physical force. This force can be transferred to the cells embedded in the scaffold, driving the tenogenic differentiation of the stem cells [13, 86]. Tomás et al. applied this strategy to cultivate engineered tendons under a magnetic field of 1.5 mT, and observed high expression of *SCX* and *TNMD* while the genes of other lineages were suppressed [13]. The upregulation of anti-inflammatory markers was also found during the process [13].

In summary, magnetic force stimulation has several advantages compared with other types of mechanical stimulation. (1) Magnetic force stimulation can regulate the inflammatory response, and therefore obtain the engineered tendon with better biological performance. (2) Magnetic force stimulation can be remotely and easily adjusted by changing the magnetic field, even *in vivo*. (3) Through MNPs, or target activated receptors, magnetic force stimulation can more effectively deliver mechanical stimulation to seeded cells.

### 6.2. The Combination of Mechanical and Chemical Stimulations

Various growth factors have been widely applied to tendon tissue engineering, and some of them have been shown to promote tenogenic differentiation and tendon regeneration. For instance, the signal pathway mediated by TGF-*β*1 is considered to be the most important signal transduction pathway that induces tenogenic differentiation. CTGF contributes to the structural integrity of tendon tissue [42]. In addition, GDF-5/6/7 has been reported to promote the expression of tendon-related genes [87–89]. Researchers usually discuss the effects of mechanical stimulations and growth factors on tenogenic differentiation separately, but each of them alone is not sufficient to get a satisfactory engineered tendon. Some growth factors are important for tendon repair and regeneration, even throughout the entire process, such as TGF-*β* [15]. We cannot guarantee that mechanical stimulation will replace the effect of growth factors or that mechanical stimulation will induce similar effects as growth factor. However, mechanical stimulation can be a good supplement to growth factors, especially for the promotion of tendon formation. It might also be that mechanical stimulation will affect the expression pattern of receptors on the cells, thus increasing or decreasing the response of certain growth factors. For example, the decreased mechanical loading downregulates the expression of TGF-*β* receptors and thus suppresses the TGF-*β*/Smad signaling pathway which is significant for tenogenic differentiation [90]. Therefore, it is of great importance to explore the synergistic combination of mechanical stimulation and growth factors.

There are a few studies that have demonstrated the synergistic effect of mechanical stimulation and growth factors like TGF-*β*1 and BMP-12 (GDF-7) in tendon tissue engineering (Table 3). Zhang et al. discovered that dynamic stretching and TGF-*β*1 synergistically increased cell viability and the expression of tendon-related genes (*Col 1a1*, *Col 3a1*, *TNC*, *SCX*, and *TNMD*) as well as their corresponding proteins [35]. Interestingly, their synergistic effects are not manifested in copromotion, but instead seem to make up for each other's negative effects. For instance, Zhang et al. found that the dynamic stretching could suppress the cell death induced by TGF-*β*1 and that growth factor inhibited the increase of the average porosity and pore size caused by cyclic stretching, which improved the mechanical properties of the engineered tendon [35]. Testa et al. demonstrated that the combination of biochemical and mechanical stimulation could synergistically promote tenogenic differentiation, resulting in an abundant and aligned type I collagen [15]. Besides, Rinoldi et al. constructed the cell-loaded highly aligned hydrogel yarns and cultured them under 15% static stretching during simultaneous exposure of BMP-12 for 7 days. They found that the synergistic action promoted the upregulation of *SCX* and *TNMD*, inducing the tenogenic differentiation of human BMSCs. Nevertheless, they also found that the expression of COL I and III were inhibited, which seemed to be related to the early differentiation and apoptosis caused by excessive strain [23]. However, not all the combinations of mechanical stimulation and growth factors can synergistically promote tendon differentiation. Farng et al. cultured the engineered tendon under static or cyclic stretching (10% strain, 0.33 Hz) during simultaneous exposure of GDF-5 for 48 h. They found that both the mechanical stretching and GDF-5 alone increased *COL I* and *SCX* expression, while additional synergistic effect of them was not observed [53]. It is possible that one of the stimulations was dominant and covered the effect of the other on inducing tenogenic differentiation [18]. In addition, the types and parameters of mechanical stimulation, as well as the concentration and delivery time of growth factors, may affect the performance of the synergistic effect on inducing tendon differentiation. During tendon repair or regeneration, growth factors may play a role at only certain times or phases and therefore dynamic release of growth factors or a stepwise-treated strategy may have better effects [18]. It requires further understanding of the mechanisms about how growth factors regulate tendon differentiation. In conclusion, the combination of different mechanical and biochemical stimulus is a potential strategy to superiorly construct an engineered tendon; however, the optimal set-up needs to be further explored.

## 7. Conclusion

Mechanical stimulation is an important regulatory factor in tendon tissue engineering, which can induce the differentiation of stem cells into tenocytes and improve the performance of engineered tendon constructs. Dynamic uniaxial stretching simulates the biomechanical conditions of natural tendons by inducing tenogenic differentiation, alignment of cells and ECM, and promotes tendon repair and regeneration. The frequency of an effective protocol in bioreactors is usually not higher than 1 Hz, and between 1% and 12% strain with sufficient rest intervals. However, an optimal protocol is dependent on other conditions as well, such as cell types and loading systems. Natural mechanical stimulation *in vivo* facilitates the formation of a more mature tendon-like tissue in the engineered tendon construct. Although some progress has been made in studying the mechanisms of mechanical stimulation to activate tenogenic differentiation, this process is still not fully elucidated and needs further investigation. In addition, a future potential in the field of tendon tissue engineering could be the development of magnetic force, as well as combining mechanical and biochemical stimulation to obtain the synergistic effect to facilitate the development of the ideal engineered tendons.

## Figures and Tables

**Table 1:** Dynamic uniaxial stretching used in tendon tissue engineering.

Cell type	Parameters	Effects	Ref
Human BMSCs	1% strain; 1 Hz; 30 min/day.	Maintained the expression of SCX.	[44]
Rat BMSCs	2% strain; 0.5 cycles/min; 30 min/day.	Increased cellularity and tensile strength; promoted ECM deposition and fiber alignment.	[24]
Rat BMSCs	2% strain; 0.5, 1, and 2 cycles/min; 0.5, 1, and 2 h/day.	Significantly increased cellularity and tensile strength; further ECM deposition and fiber alignment.	[43]
Rabbit TDSCs	2% strain; 1 Hz; stretching and rest alternated.	Promoted tenogenic differentiation (COL 3A1 and DCN).	[37]
Rat BMSCs	2.4% strain; 1 Hz; stretching for 20 s and resting for 100 s.	Significantly promoted *COL I* expression; increased stiffness of construct.	[45]
Rabbit MSCs	2.4% strain; 1 Hz, 8 h/day.	Significantly increased *COL I* expression. Increased the linear stiffness of construct.	[46]
Dog BMSCs	3.0% strain; 0.2 Hz; 20 min/h, 12 h/day.	The elongated cell morphology; promoted cell infiltration and retained mechanical properties; promoted the tenogenic differentiation.	[7, 47, 48]
Equine BMSCs, ASCs, TDSCs	3% strain; 0.33 Hz; 1 h/day.	Promoted cell infiltration and tenogenic differentiation; increased mechanical properties.	[49]
Human ASCs	4% strain; 0.5 Hz; 2 h/day.	Significantly increased the tendon-related genes and proteins.	[33]
Rabbit BMSCs	A 5% translational strain and a 90° rotational strain; 0.1 Hz; 12 h/day.	Upregulated the expression of tendon-related ECM proteins (COL I, TNC, and TNMD); promoted cell alignment.	[50]
Human BMSCs	5% strain; 1 Hz; 1 h/day.	An upregulation in a number of key tendon genes (*Col1a1*, *Col1a2*, *Col3a1*, *TNC*, *ELN*, and *FN*).	[51]
Rat TDSCs	6% strain; 0.25 Hz; 8 h/day.	Induced tenogenic-specific differentiation; aligned and compact F-actin network.	[10]
Human fibroblasts	10% strain; 0.25 Hz; 8 h/day.	Significant increased cell proliferation and increased *COL I*, *TFG-β1*, and *CTGF* expression; increased COL I and FN deposition.	[52]
Human BMSCs	10% strain; 0.33 Hz.	Increased *COL I*, *COL III*, and *SCX* expression compared to control group.	[53]
Murine fibroblasts	10% strain; 0.5 Hz.	Better alignment of collagen fibers and proper organization of ECM.	[15]
Human BMSCs	10% strain; 1 Hz; 2 h/day.	Enhanced expression of *COL I*, *EphA4*, and *SCX*; elongated cell morphology.	[34, 54]
Human BMSCs	10% strain; 1 Hz; 3 h of strain followed by 3 h rest.	Significantly upregulated tendon related genes (*COL I*, *COL III*, and *TNC*).	[55]
Human BMSCs	10% strain and axial rotation; 1 Hz.	Significantly enhanced cell infiltration, matrix synthesis (COL I and III and TNC), and ultimate tensile load of engineered tendons.	[8]
Equine BMSCs	3%, or 5% strain; 0.33 Hz; 1 h/day.	3% strain promoted cell infiltration, tenogenic differentiation, and increased construct elastic modulus and ultimate tensile strength.	[56]
Human BMSCs	4, 8 or 12% strain; 0.5 or 1 Hz.	The highest proliferation rate at 1 Hz and at 4% strain. The highest tenogenic expression at 8% and 12% strain.	[27]
Human BMSCs	4, 8 or 12% strain; 1 Hz.	Higher tenogenic gene expressions at 8% (highest) and 12% strain (COL I, COL III, FN, and N-cadherin).	[28]
Rabbit TDSCs	*In vitro*: 4% strain; 0.5 Hz; 2 h/day. *In vivo*: implanted into the mouse back.	*In vitro*: promoted tendon-specific genes and protein expression. *In vivo*: more parallelly arranged matrixes (COL I, COL III, and TNC); the mature engineered tendon. Both: increased cell proliferation, elongated cell morphology, and mechanical properties.	[6]
Human ESC-MSCs	*In vitro*: 10% strain; 1 Hz; 2 h/day. *In vivo*: implanted into the mouse back.	*In vitro*: unregulated the expression of tendon-related genes *(SCX*, *COL I*, *COL III*, and *Epha4*). *In vivo*: elongated morphology of cells; promote more parallel alignment.	[57] [58]
Rat BMSCs	*In vitro*: 15% strain; 1 Hz. *In vivo*: implanted into the hind limbs of mice.	*In vitro*: increased cell viability and the expression of *SCX and TNC*; COL1a1 and TNC expression did not significantly change; increased pore size. *In vivo*: better mechanical properties and cell alignment (after prestretching *in vitro*).	[35]

**Table 2:** Static uniaxial stretching used in tendon tissue engineering.

Cell type	Parameters	Effects	Ref
Equine ASCs	4% strain; stretching for 2 h and followed by a 6 h pause.	Promoted cell alignment; more spindle-shaped cell and the elongated nucleus.	[64]
Human BMSCs	15% strain (day 0-7); 30% strain (after 7 days).	Enhanced COL I and III expression and its alignment; promoted *SCX*, *TNMD* expression. Promoted cell adhesion, alignment, and proliferation.	[23]
Human dental pulp stem cells (DPSCs)	Maximum tensile force (just below the failure load).	Expressed *COL I* and *VI* but rarely expressed tendon-related proteins.	[65]
Human BMSCs	Double the length of the construct.	The packed and aligned fibrils. Increased ultimate tensile stress.	[66]
Human BMSCs	The constant tension generated by a bioreactor.	Upregulated the expression of SCX; modulated elastin and COL III, XII, and XIV expressions.	[44]
Human fibroblasts	The constant tension generated by a U-shaped spring.	Production of fibers of COL I & III that were aligned longitudinally.	[67]

**Table 3:** Combination of the mechanical and biochemical stimulations in tendon tissue engineering.

Cell type	Biochemical stimulation	Mechanical stimulation	Effects	Ref
Murine fibroblasts	5 ng/ml TGF-*β*1.	10% strain; 0.5 Hz.	Synergistically promoted the tenogenic differentiation.	[15]
Rat BMSCs	10 ng/ml TFG-*β*1.	15% strain; 1 Hz.	Synergistically increased cell viability, the tenogenic differentiation, and the mechanical properties of construct.	[35]
Human BMSCs	10 ng/ml BMP-12.	Static tension (day 0~7: 15% strain; day 7~: 30%)	Synergistically promoted the tenogenic differentiation and cell alignment.	[23]
Human BMSCs	hGDF-5/BMP-14 (loaded into the PLGA microcarriers).	10% strain; 1 Hz.	Synergistically induced the expression of COL I and III, DCN, SCX, and TNC.	[34]
Equine ASCs	10 ng/*μ*l GDF-5,6,7.	4% strain; stretching for 2 h; followed by a 6 h pause.	Induced the higher tendon associated gene expression, especially for COMP and SCX compared single stimulus.	[64]
Rat BMSCs	1600 ng/scaffold GDF-5.	10% strain; 0.33 Hz.	Mechanical stimulation and GDF-5 increased the expression of *COL I* and *SCX* compared to control. No obvious additive synergism.	[53]
